# Living well to the end: A phenomenological analysis of life in extra care housing

**DOI:** 10.3402/qhw.v11.31100

**Published:** 2016-05-10

**Authors:** Rachel L. Shaw, Karen West, Barbara Hagger, Carol A. Holland

**Affiliations:** 1 School of Life and Health Sciences, Psychology Department, Aston University, Birmingham, UK; 2 School of Language and Social Sciences, Sociology and Policy Department, Aston University, Birmingham, UK; 3 Aston Research Centre for Healthy Ageing (ARCHA), ARCHA is the Department, Aston University, Birmingham, UK

**Keywords:** Ageing, extra care housing, well-being, lifeworld-led care, phenomenology, qualitative research

## Abstract

**Objectives:**

To understand older adults’ experiences of moving into extra care housing which offers enrichment activities alongside social and healthcare support.

**Design:**

A longitudinal study was conducted which adopted a phenomenological approach to data generation and analysis.

**Methods:**

Semi-structured interviews were conducted in the first 18 months of living in extra care housing. Interpretative phenomenological analysis was used because its commitment to idiography enabled an in-depth analysis of the subjective lived experience of moving into extra care housing. Themes generated inductively were examined against an existential–phenomenological theory of well-being.

**Results:**

*Learning to live in an extra care community* showed negotiating new relationships was not straightforward; maintaining friendships outside the community became more difficult as capacity declined. In *springboard for opportunity/confinement*, living in extra care provided new opportunities for social engagement and a restored sense of self. Over time horizons began to shrink as incapacities grew. *Seeking care* illustrated reticence to seek care, due to embarrassment and a sense of duty to one's partner. *Becoming aged* presented an ontological challenge. Nevertheless, some showed a readiness for death, a sense of homecoming.

**Conclusions:**

An authentic later life was possible but residents required emotional and social support to live through the transition and challenges of becoming aged. Enhancement activities boosted residents’ quality of life but the range of activities could be extended to cater better for quieter, smaller scale events within the community; volunteer activity facilitators could be used here. Peer mentoring may help build new relationships and opportunities for interactive stimulation. Acknowledging the importance of feeling—empathic imagination—in caregiving may help staff and residents relate better to each other, thus helping individuals to become ontologically secure and live well to the end.

Ageing, or becoming aged, has been described as a “fundamental ontological challenge” (Gilleard & Higgs, 2010, p. 125). This paper explores the lived experience of ageing in extra care housing. It presents findings of qualitative research undertaken as part of a longitudinal mixed-methods study (Holland et al., 2015). Our objective was to examine individuals’ subjective experiences of moving into and living in extra care housing. Extra care has the “aim of meeting the housing, care and support needs of older people while helping them maintain independence in their own private accommodation” (Netten, Darton, Baumker & Callaghan, 2011, p. 4). Extra care housing aims to enable residents to remain physically and mentally active, independent and socially engaged. The “problem” of ageing has become a central concern of health and social care institutions (Dahlberg, Todres & Galvin, 2009; Todres, Galvin & Dahlberg, 2006) fuelled by the dividing practices of the idealization of “active” or “successful” ageing in what has been called “the third age” (Baars, 2012; Gilleard & Higgs, 2010; Katz, 2000). Consequently residential care homes can be seen as a negative lifestyle choice (Higgs & Gilleard, 2015): retirement communities like extra care housing, however, offer care and support while maintaining independence, rationality, and agency.

We adopted a phenomenological approach to health psychology which is committed to humanizing healthcare by bringing together the subjective lifeworld of the person within the systems approach of health and social care institutions (Walseth & Schei, 2011). This “view from the inside” enables a holistic understanding of lifeworld-led care (Dahlberg, Todres & Galvin, 2009; Todres, Galvin & Dahlberg, 2006) and how it may be created in older age. According to Husserl, the lifeworld (*Lebenswelt*) is the start and end point of human existence; it is “the universal framework of human endeavour—including our scientific endeavours. It is the ultimate horizon of all human achievement.” (Moran, 2000, p. 181–2). The problem for us as researchers, and as people, is that this “pre-given” lifeworld can become obscured as we take for granted the theories and postulations of the “natural attitude.” Instead, Husserl called for the adoption of a “phenomenological attitude” which “involves describing the world the way it is experienced by humans; what the world is and means to humans, what it means for humans to have a world, and how humans relate to this world, to each other, to different situations—to all possible “things” of the world.” (Dahlberg, Dahlberg & Nyström, 2008, p. 36). The lifeworld has been further elaborated as a set of interrelated dimensions which can help describe and understand it in a way that approximates its wholeness: spatiality, temporality, intersubjectivity, mood, identity, and embodiment (Todres et al., 2006; further writings about the various interpretations of the elements of the lifeworld can be found in: Boss, 1979; Husserl, 1936 [1970]; Heidegger, 1927 [1962]; Merleau-Ponty, 1945 [1962]). For the purposes of this study, this means that we need to gather in-depth experiential accounts and to meet with people in their “home-world” over a period of time for us to reveal the significance of the environment on their sense of well-being. By focusing on the elements of the lifeworld we are able to engage in an analysis of those subjective worlds within the systems world of the health and social care landscape.

If elements of the lifeworld change due to a change in circumstance, it is likely to be anxiety-provoking. Moving into new accommodation with the provision of “extra care” may be experienced as one such anxiety-provoking, ontological—or existential—challenge. For Heidegger, anxiety reveals a sense of homelessness, a feeling that the rug has been pulled out from under us (Moran, 2000). The same could be argued in relation to our bodies: when well, we feel at-home in our bodies, but when illness or incapacity strikes we begin to feel “disharmony, dis-equilibrium, dis-ability, and dis-ease which incorporates a loss of the familiar world” (Toombs, 1993, p. 96); a sense of existential homelessness (Todres & Galvin, 2010). This resonates with Bury's (1982) notion of biographical disruption and is a familiar theory within illness stories (for a review, see: Smith, 2011). It also reminiscent of the theory of posttraumatic growth (e.g., Boyraz & Efstathiou, 2011). Indeed, Heidegger's theorem is that existential homelessness creates an energizing potential which could be experienced as well-being. Heidegger believed that most of the time we live inauthentically and go along with the crowd without thinking about our innermost, “ownmost” being (Moran, 2000). Nevertheless, he theorized that central to our being was a sense of mobility, a movement toward a “potential-to-be-whole”; thus, living an authentic life is possible when we are able to gather ourselves and move toward this wholeness (Moran, 2000). Heidegger proposed that the route toward authentic homecoming, and thus an authentic later life, comes through existential homelessness (Todres & Galvin, 2010). To live an authentic life is to be ontologically secure, i.e., to embody a stable sense of self, to feel at-home, and to be imaginatively engaged in society in ways that are meaningful to the individual and offer opportunities for living well or well-being (Baars, 2012; Galvin & Todres, 2013; Phillipson & Biggs, 1998).

Galvin and Todres’ (2013), Galvin and Todres’ (2011) theory of well-being follows Heidegger's (1927 [1962]) proposal of human existence as oriented toward future possibilities and “being-at-home-with” what has been given. This is expressed by the term, dwelling-mobility, which is signified by the presence of both future possibilities and at-homeness; dwelling-mobility thus offers the deepest possibility for well-being which brings with it “a feeling of rootedness and flow, peace and possibility” (Galvin & Todres, 2013, p. 65). Using this existential–phenomenological theory different opportunities for well-being can be delineated by examining the essential features of the lifeworld which represent dimensions of human experience. In this study we adopted Galvin and Todres’ (2011) existential–phenomenological theory of well-being as a sounding board for making sense of older adults’ experiences of living and ageing in an extra care community (see Table I).

**Table I:** “Dwelling-mobility lattice” adapted from Galvin and Todres’ (2011) theory of well-being.

Element of the lifeworld	Mobility	Dwelling	Dwelling-mobility
Spatiality	Adventurous horizons	At-homeness	Abiding expanse
	Anything that offers a place of promise. A sense of adventure where spatial possibilities arise that offer movement (metaphorically or literally).	A sense of being at-home, feeling of being settled/still within the physical environment that is wanted. Familiar and comfortable surroundings (favourite easy chair) or having familiar/personal objects close-by.	Both a feeling of at-homeness with possibilities for adventurous horizons. Being deeply connected to a place but also having a stepping out point to go further afield (metaphorically or literally).
Temporality	Future orientation	Present-centredness	Renewal
	Being energized by future possibilities (metaphorical or literal) which emphasize the sense of flow, not being stuck.	Absorbed in the present in a way that is desired. Sense of belonging, being “in the zone.”	Unification of future possibilities with a satisfaction with the now. Rooted flow—a sense of being absorbed in the now and a welcome readiness for the future.
Intersubjectivity	Mysterious interpersonal attraction	Kinship and belonging	Mutual complementarity
	In tune with interactional possibilities and an attraction to people's “otherness,” understanding the mystery of others.	An effortless being together; “we” rather than “I” and “you.”	Both a sense of kinship/togetherness and excitement at learning new things—a “homelike oneness” and difference.
Mood	Excitement or desire	Peacefulness	Mirror-like multidimensional fullness
	Sense of “attunement” and buoyancy of movement (looking forward to a longed-for holiday or special event).	Stillness, settledness. There is a welcomed pause, coming to accept things, and “letting be.”	Complex mood encapsulating the energy of moving forwards and a sense of being at one with the world and oneself.
Identity	“I can”	“I am”	Layered continuity
	Sense of being able to. Experiencing oneself as being on the move (literally or metaphorically).	A sense of self that is supported by continuous histories and contexts that fit with who “I am.”	A continuous sense of “I can” and a strong sense of “just being” in a foundational sense. Ontological security.
Embodiment	Vitality	Comfort	Grounded vibrancy
	Tuned into an embodied energy that offers the possibility of movement, “bodying forth.”	Literal feeling of comfort, warmth, relaxed, satiated. Felt sense of familiarity and intimacy with one's body.	An energized flow and a bodily sense of feeling deeply at-home. Both “being” and “becoming” is possible.

## Method

### Setting

This study was set in one UK organization, *ExtraCare Charitable Trust* (henceforth, ExtraCare). ExtraCare communities are a mix of large (c.400 residents) purpose-built “villages” of self-contained apartments and smaller sites (c. 50 residents), converted from existing assisted living housing, organized around a central hub including communal facilities (e.g., leisure and fitness facilities, café or restaurant, hobby rooms, grocery store), a drop-in clinic with an on-site well-being advisor (a nurse), support for prevention involvement such as falls prevention, “locksmith” services for those experiencing cognitive difficulties (Brooker & Woolley, 2007), and on-site security. Village residents are on average 10 years younger than those in smaller sites (average of 74 and 84 years old respectively). Services include personal care, companionship, and housekeeping. Support allocating private or local authority funds to pay for care is available. All sites offer a programme of entertainment and enrichment activities.

### Participants

Ethical permission was granted from the University Ethics Committee to recruit participants in collaboration with representatives from ExtraCare. Six residents were recruited (see Table II).

**Table II:** Demographic data for participants.

Pseudonym (Gender)	Year of birth	Ethnicity	Marital status	Time in ExtraCare housing at first interview	Size of ExtraCare site	Reported diagnoses	Reported services received
Annie (F)	1949	White British	Divorced	4 months	Large	Arthritis, respiratory condition	Private care
Clive (M)	1930	White British	Widowed	2 months	Small	Arthritis, age-related macular degeneration	Care level 2, social support
Derek (M)	1932	White British	Divorced	5 months	Small	Age-related macular degeneration	Social support
Edgar (M)	1931	White British	Married^a^	5 months	Large	Stroke	Cared for by wife (Eleanor)
Eleanor (F)	1935	White British	Married	5 months	Large	Chronic pain, hypertension	None
Martha (F)	1930	White British	Widowed	2 months	Small	Chronic obstructive pulmonary disease	Care level 5, social support

a Edgar was married to Eleanor.

### Data collection

In-depth semi-structured interviews were conducted at three time points: within five months of moving in, at 12 and 18 months after moving in. Initial interviews focused on moving in: reasons for moving in, use of facilities, involvement in organized activities, and uptake of care; second interviews discussed settling in, changes since moving in, any others matters relating to living in ExtraCare, seeking care, or well-being; final interviews followed-up the same issues again. Interviews were audio-recorded and transcribed verbatim. Interviewing participants more than once enabled an in-depth biographic approach. Furthermore, in later interviews we were able to examine in-depth concepts that had been raised previously to establish a detailed and sensitive understanding of participants’ lived experience.

### Analysis

Interpretative phenomenological analysis (IPA; Smith, Flowers & Larkin, 2009) was used because of its commitment to idiography and its focus on revealing participants’ own meaning-making of their experiences. Previous research has focused on a societal understanding of extra care housing whereas in this study the goal was to come to understand how it is experienced by individuals and how they make sense of themselves and their world when that world is extra care housing (Grenier & Phillipson, 2013). The analysis followed Smith et al.'s (2009) guidance. Notes were made highlighting evocative language use (imagery, metaphor), clues to positionality of the individual (self-concept, significant relationships), and matters of concern which featured strongly within the account. A summary for each case was written for each time point. Key themes were generated from individuals’ accounts and compared over the different points in time. We then examined themes against Galvin and Todres’ (2013) dimensions of well-being (Table I); this involved a dialogue between data gathered and well-being theory, thus adopting a process of abductive reasoning (e.g., Hiles, 2014) or inference to the best explanation (Harman, 1965). This was a cyclical process between inductive themes generated and theoretical dimensions of well-being. Discussions between authors ensured transparency of meaning-making and that interpretations were evidenced in the data. Engaging in “thinking dialogue” about the data during analysis facilitated an openness to seeing something new from the different perspectives of analysts, drawing on the process of hermeneutic interpretation embraced by IPA and reflective lifeworld research (Dahlberg, Dahlberg & Nyström, 2008). This reflexive approach produced a self-aware and dialogic process (Shaw, 2010).

## Results

Themes are presented in turn using data extracts (see Table III).

**Table III:** Themes generated in the analysis with details from each participant's account.

Theme	Element of the lifeworld	Notes	Participants contributing to this theme
Learning to live	Intersubjectivity	• Social circle—new and existing	• Annie, Derek, Clive
in an extra care		• Resistance/aversion/politics	• Annie, Clive
community		• Mysterious interpersonal attraction	• Derek
		• Living vicariously through others	• Edgar
Springboard for	Spatiality	• Being able to get around/out	• Annie
opportunity/		• Previously isolated, caged bird set free	• Eleanor, Annie
confinement		• Links to social circle/existing friends/new friends	• Annie, Eleanor, Derek, Clive
		• Larger cage, these four walls	• Eleanor, Clive
		• World shrinks but that's okay	• Derek
	Temporality	• Blocked future/elusive present	• Eleanor, Annie
		• Fear of the future, being trapped	• Annie
		• Inability to see a better/different future	• Eleanor
Seeking care	Embodiment	• Vitality despite incapacity, being cared for	• Martha
		• Resisting care	• Annie, Eleanor
	Identity	• Being an object/thing	• Annie
		• Letting be/letting go	• Edgar, Martha, Derek
	Mood	• (Initial) peacefulness	• Eleanor, Annie, Clive, Martha
		• Later agitation	• Annie, Clive
Becoming aged	Embodiment	• Adjusting to incapacities	• Derek, Clive, Annie, Martha
		• Overwhelming fatigue, pain	• Annie, Clive, Derek
	Identity	• Readiness for death	• Edgar
		• Ontological security	• Edgar, Martha
	Mood	• Acceptance/at peace	• Edgar, Derek

### Learning to live in an extra care community

Participants had moved from their family home either directly to ExtraCare or via a hospital stay. Some remained in the same geographic location (Martha, Derek, Annie, Clive^1^) but Eleanor and Edgar, a married couple, had moved location to be close to family. Most had existing friendships close-by; Annie and Derek made conscious efforts to retain those relationships. Geographical space was important for some to maintain a sense of intersubjective self through existing social relationships. This was the case for Annie, whose existing sources for social interaction and engagement helped sustain her values and sense of self.

I don't want to get tied into anyone here particularly because living here with them if they're not my cup of tea shall I say I would feel very worried and anxious about the fact that I'm tied into that relationship and might not want to be. So I'm very much making a conscious decision that I will be friendly with everyone but not to sort of forge a great relationship because I'm in the position that I don't feel lonely that I've got a good circle of friends that I don't need to look for another friendship really. (Annie, Interview 1)

Annie explained that her religious background meant she was teetotal and hearing residents blaspheme made her uncomfortable. This conjures images of other types of closed communal, institutional living where rooms, corridors, and thus neighbours are fixed illustrating how spatiality impacts on intersubjectivity and identity. For Annie, the physical space in which she lived posed a threat to how she made sense of who she was through interactions with others in her social world. Later in that first interview, Annie described an interaction with another resident who, to her surprise, had understood Annie's likes and dislikes and suggested meeting in the coffee bar instead, Annie reported saying to this resident, “you're either very astute or you realize I didn't say yes.” Annie was pleased that this individual had plenty of friends and relatives to keep her otherwise engaged. Annie was relieved that nobody was going to “coerce” her into taking part in community life and said, “that suits me fine.”

In her second interview Annie was beginning to form relationships: she described her neighbour as “a little gem” but being able to spend time alone remained important to her: “my way of life now is very sociable, I mean I've even got brave enough to go and eat somewhere on my own. So I wouldn't have done that before” suggesting that living in ExtraCare facilitated her growing self-confidence. She described her way of life as “lazy, laid back […] a lovely way of life” intimating that she is able to relax because other things are taken care of.

By her third interview, Annie talked about the activity group she had joined with stories of a “cantankerous woman” who “upsets everyone.” Martha had a similar experience.

There were one [lady] when I first come. The ladies were playing on the first night. […] The first time I went into play she got them to take her straight back. I thought well, I said “I ain't come to take her place, I don't mind watching.” […] The [staff] told me off when I said about it. They said “don't you ever feel like that, you're pushing nobody out at all.” (Martha, Interview 1)

This indicates the length of time needed to build up relationships and the community politics bound up within joining organized activities. Through the staff's intervention, Martha was able to retain a sense of her past self through engaging in social interactions within the context of activities she found enjoyable and which made her who she was.

Derek also lived in a smaller ExtraCare site. Like Annie, he maintained existing friendships and social engagements: “Everybody laughs at me when I get a taxi to the [sports] club every Saturday afternoon. […] All I do is go and have a couple of beers with old friends and reminisce.” He went to the local pub, initially walking but later by a taxi. Socializing was important to Derek. He explained how his working life had taken him all over the world, meeting interesting people and experiencing different cultures. This fascination for others helped Derek settle into ExtraCare. Other residents were living with health conditions with which he was unfamiliar but he showed the same intrigue about his fellow residents’ lifeworlds as he did when travelling overseas. He found talks hosted by ExtraCare very useful.

[staff member] is now giving these talks in the afternoons, which I've found very informative […] the talks are aimed at us 80 years plus, puts me in context, gives me an understanding of what other people are talking about. I want a better understanding of what goes on […] It was fascinating really. […] Knowledge is what makes you more comfortable, knowing what the hell is going on. (Derek, Interview 1)

Social engagement made Derek feel at-home with himself because this was how he had always been. However, deteriorating sight severely affected Derek's opportunities for social interaction and his intersubjective self: “I have trouble recognizing faces […] I try very hard but I don't recognize half of them still. […] But there seems to be a general turnover of people” (Interview 2). The turnover of residents, who Derek says either died or were “shipped to hospital” didn't help. By his third interview, Derek felt excluded from the community because he couldn't recognize people.

There's probably half a dozen people who've come within the last few months that I've never met and some of them if I get close enough are peering at me and they're “why doesn't he say anything?” You can see their thoughts on their faces. Because they expect the men to introduce themselves first don't they. (Derek, Interview 3)

This hit Derek hard: his lifeworld had been filled with social encounters and as this slipped away, his relational self as social actor began to fade too and the buoyancy of his earlier interviews reduced. Derek's intersubjective self was threatened by his vision loss, and he saw no way of adapting his social world. Social engagement is a key benefit of extra care housing with good reason. However, those communities are made up of microcosms of wider society and so a person's readiness or ability to engage socially should not be taken for granted. Residents may need assistance to fully engage for as long as desired despite increasing incapacity. Furthermore, this support may be needed for an extended period of adjustment not just immediately on moving in.

### Springboard for opportunity/confinement

The physical environment of ExtraCare sites played a significant role in the lives of participants illustrating the importance of spatiality when growing older. For Annie, who had experienced mobility problems for most of her life, it was simply being able to access everything: “being on one level is a boon!” For Eleanor, her new environment gave great opportunity for social interaction and a renaissance of her past self. Prior to moving into ExtraCare, Eleanor described herself as a “caged bird.” Her husband, Edgar, had a stroke a year previously and she had been his main carer since forcing her to withdraw from her social circles. It was clear that Eleanor loved her home and took pride in being a homemaker. When they sold their house to move to ExtraCare, Eleanor was devastated by the subsequent re-development of her former home.

We left it immaculate […] They've ripped everything up, the garden, everything. Edgar said to me “leave it, gone, it's gone, leave it.” But I can't. […] And he said “done, it's finished.” That's our life gone, that's part of our life gone. But that was my life. I mean, I've been there fifty four years, my husband was there since he was about eight. (Eleanor, Interview 1)

Eleanor's lifeworld was physically and emotionally tied to her home; her husband thought she was being over-sentimental but the way she felt about leaving her home was clear. Eleanor's sense of at-homeness was spatially within the building she called home. Eleanor's conceptualization of living in ExtraCare as like being on holiday is illuminating in this context.

To me, it's been fantastic. It's just like being on holiday! [and considerably later] It is like being on holiday. Do you know, it reminds me of a cruise ship here. […] And this is like your cabin and you come out you see and you've got everything there for you. I mean you've got the rest. I mean on a cruise ship you can eat 24 hours a day there. But here, if I don't want to cook we can go to the restaurant, it's a beautiful restaurant. (Eleanor, Interview 2)

The cruise ship metaphor represents both Eleanor's sense of adventure and the peacefulness she feels on holiday. However, that image of the cage isn't too far away; representing her apartment as a cabin with all amenities on board reminds us that on a cruise ship going overboard is not recommended. Reminiscent of Annie's reticence to establish new relationships; there is no obvious means of escape. Place and space were significant to Eleanor, which is emphasized by the fact that she moved location when moving into ExtraCare. This meant she felt anxious about leaving the village because she didn't know her way around. Instead, Eleanor satisfied herself with walking around the grounds. But when she spoke of this, speculations about Eleanor's confinement in ExtraCare came to the fore: “I've got to get out in the fresh air, *out*, because you can get quite drawn-in here” (Interview 2). Eleanor emphasized the word, “*out*.” Ostensibly happy in ExtraCare, Eleanor's sense of well-being was under threat because she felt confined. Part of this confinement was due to her caring duties for her husband (which should not be underestimated and will be covered later) but there was an evident appetite for more freedom and open space that Eleanor missed which didn't dissipate over time.

I would like just one more cruise. (Eleanor, Interview 2)

We're inside a lot, I do like to do a little walk about each day, out in the fresh air. I go around the perimeter here. (Eleanor, Interview 3)

Clive's sense of space also changed over time. In his first two interviews Clive said he had settled well into his apartment after lengthy hospitalization and was happy. By the third interview he described being less able to care for himself and was troubled by illness. This interrupted his lifeworld and he began to feel trapped.

I love this place and I love the garden and I love the flat, but you're in between the same four walls, day in day out, 24/7 and it's, I go nowhere, I can't go nowhere you see. […] [singing] Four walls to see, four walls closing in on me. Like they do, they close in on you. (Clive, Interview 3)

Music was important to Clive and during the time spent with us he played some of his favourite songs. While listening to the music he sang along and seemed to be transported to another place, another time. However, Clive told a story of an incident between him and others living in ExtraCare following which he withdrew from organized activities (“that's why I can't join in any activities”) removing opportunities for social interaction because he could no longer go out alone (after falling on a previous outing, he had lost his confidence). It was clear that this was particularly challenging for him.

I'm vulnerable now. But I feel that I'm going to prove to them [the other residents/staff] without forcing it, without looking for it, I'm going to prove to them that if they come the acid with me I'm going to let them know that I'm not to be messed about with. (Clive, Interview 3)

This shows Clive exerting his masculinity in the only way that was left; in his own words, he “went from a strong man to a nothing” (Interview 2) and was struggling to come to terms with his changing and weakening body. Clive drew on powerful imagery to express how this made him feel.

You know the old tripe they used to have hanging in the butchers shops? My arm drops like that […] My legs are skinny […] deterioration I suppose […] lack of activity […] I'm getting older. (Clive, Interview 3)

These extracts demonstrate the interconnectedness between spatiality, embodiment, and intersubjectivity within participants’ lifeworlds. The interplay between these elements of the lifeworld became intensified for Clive and Derek as disability and ill-health increased over time threatening their social engagement, their intersubjective self and their embodied self.

### Seeking care

Stoicism epitomized Annie's approach to personal care despite her perceiving it as an inevitability. She was very resistant about seeking help too soon, though she had researched her options and knew what was available to her.

I have health issues which are a little bit personal and I don't know that I can handle at the moment somebody doing what I might need help with and I would rather struggle on. Now that might sound like pride, it's not, it's embarrassment. […] So I'm at a bit of a crossroads I think. I would like not to have to change anything because I fiercely want to keep my independence anyway. But I feel worried about that, if I am going to deteriorate anymore and I do need some help, erm, coming to terms with having what feels a little bit invasive if that makes sense? (Annie, Interview 2)

The need for personal care was a faît accompli in Annie's mind and while rationally knowing this, it made her feel uncomfortable. In the extract above, Annie explicitly sought help with coming to terms with this shift in her life, her reticence at not wanting to feel like an object or a thing, that her sense of identity would become entangled with her failing body. Annie's fight to retain her independence had become a fight to retain her sense of self as someone other than the physical body in which she found herself.

Martha lived in a smaller site and was almost 20 years older than Annie. Martha had embraced care and received the highest level of care available and soon developed good relations with care staff. Martha's health had already deteriorated; her chronic obstructive pulmonary disease meant she had experienced dangerously low oxygen levels in the past, causing her to lose consciousness. Since then, she had been prescribed oxygen and was unable to walk. Yet she described her lifestyle as busy and spoke often about the activities she enjoyed. She particularly enjoyed being taken to these events; the care and assistance Martha received facilitated her social life and there was no sense of stigma or hurt pride.

That's the best bit about it, I ain't got to go far. […] They come and fetch me and fetch me wheelchair in and bring me back and pack it away again. […] we got it all laid on […] I couldn't have enough done for me […] and I come back full of beans because I've done it all even with no trouble. […] I'd recommend it to even the Queen couldn't be treated better. (Martha, Interview 2)

The fact that someone was always “at the end of the [emergency] cord,” relieved Martha's anxiety. She felt safe in ExtraCare which brought a sense of peace that enabled her to forget her anxieties and begin to enjoy her leisure time.

All of my books that I'd never read. When I moved here, I read all of them. (Martha, Interview 2)

For Edgar's wife and main carer, Eleanor, things were different. Eleanor's life had changed since moving into ExtraCare but she felt restrained by her sense of duty to Edgar, a duty which would not be relieved by others taking on day-to-day caring duties; the responsibility for Edgar's health would remain with her. Indeed, by the third interview, Eleanor spoke openly about the guilt she felt when she considered seeking help caring for Edgar (though she had accepted help elsewhere: “I now have a cleaner which is lovely” Interview 3).

I've got the guilt feeling all the time. […] I'd feel guilty if I wasn't looking after Edgar. But I do know that if I couldn't, I would be absolutely sensible and pay for whatever we could afford to have. (Eleanor, Interview 3)

There was a sense in Eleanor's third interview that she had already reached the point when support would alleviate her anxieties about Edgar.

Eleanor:Edgar is always saying to me, “you're doing too much, you're doing too much” but I do what I feel I can do then when it gets too much for me I won't do it because as I say I'm doing [a show] now. I'm also in the choir here. […]

Interviewer:How do you feel [to Edgar], is it nice to have some time on your own?

Edgar:I don't mind being on my own if I know where Eleanor is. […] I make myself a cup of tea.

In this third interview, Eleanor and Edgar were interviewed together and the possibility of taking up care was discussed. By this stage, Edgar was concerned about his wife and Eleanor was worried that Edgar would injure himself. For Eleanor, there was selfishness embedded within this dilemma; her newfound freedom within and beyond the ExtraCare community was the primary reason for contemplating paying for care for Edgar; she perceived this as neglecting her marital duty. Like Annie, Eleanor needed to be shown the benefits of taking this step and helped to make the transition. This reticence to seek help was bound up with the construct of independence; rather than perceiving help seeking as a facilitative step to free up time for leisure activities, like Martha did, Eleanor and Annie were locked in the mind-set that seeking help was synonymous with admitting defeat.

### Becoming aged

Derek talked a lot about ageing (“I've just got general old age” Interview 1), emphasizing the interconnections between body, identity and mood. In the first interview Derek described moving to ExtraCare because of his visual impairment; he had increasing difficulty walking but still managed to get out. The second interview took place in winter; Derek described the “cutting wind” and how he had been prevented from going out “purely because of the weather” which was causing him some concern (“it's been a long winter hasn't it” Interview 2). Derek's expression, “becom[ing] more aged, rather than getting old, aged” speaks to the notion of older age as something different, something one “transitions” into; Derek saw himself in the midst of this process. There was a sense of things shutting down in the way Derek talked.

Well getting up and getting oneself organized takes longer and longer. […] For exercise I walk the length of this corridor, go downstairs, walk the length of the bottom floor […] sometimes sit down and have a nap there. That's one of the things I've noticed the last few weeks. I fall asleep at any time of the day regardless of what I've been doing. […] I just happily go to sleep, don't know why. […] Something must be running down but I can't find out what it is […] starting to remind oneself of one's inabilities. (Derek, Interview 2)

The exhaustion Derek experienced in the interviews revealed his low energy levels (“you've about melted my brain” Interview 2; “I think my brain is addled, seriously I went blank there” Interview 3; he only reticently took rest after much persuasion from the interviewer). Becoming aged was something Derek anticipated, had accepted (“you've just got to learn to put up with things” Interview 3).

Edgar too had come to accept his agedness. He also enjoyed reminiscing about his life and travels. Although he was adamant that he didn't have regrets and dwelling on the past was counterproductive, there was an air of regret (perhaps nostalgia) in the way he spoke (“I can't sing like I used to” Interview 1). Nevertheless, Edgar emphasized the importance of accepting one's situation (“You've got to try and do the best you can with what you've got. […] You've got to look at your life and you say I'm going to make the best of that. And you do” Interview 1). Edgar was recovering from a stroke, with Eleanor's (his wife) support. He learned to speak again, used the gym and learned to shave with his weaker hand. But, Edgar was unable to join in the activities his wife was involved in. Instead he enjoyed reading and puzzles. He also gained vicarious enjoyment and a sense of well-being through Eleanor's activities and was able to engage with his neighbours and their pets (“I sit on the chairs outside, my hat on, and people come past, ‘how are you?’, you talk to them […] and the woman who has a [dog] she comes up to me, she says, ‘hello, shall I put the dog on your lap?’ and she sits there” Interview 2). These interactions, at home and at the café, his contentment at seeing Eleanor happy now she was singing again, reading and puzzles all seemed to satisfy Edgar. There was a sense of time having elapsed and that that was okay. Edgar's reminiscence of the life he had lived and the satisfaction he felt signified an acceptance of his aged sense of self.

You can't sit looking at what might have been. […] sometimes I hate it, but each chapter, it's happened. […] Think of all that time, I've had all that time, erm, good life. (Edgar, Interview 2)

The metaphor of a “good innings” comes to mind when thinking about the experiences of ageing described by Derek, Edgar and Martha. While the others were still coming to terms with ageing and what it meant for them (Clive) or were battling between an unwanted sense of dependency through seeking care (Annie, Eleanor), Derek, Edgar and Martha each evoked a quiet acceptance and, indeed, readiness for death. While Clive, Annie and Eleanor continued to fear the meaning of the fourth age for them and their identities, Derek, Edgar and Martha had arguably passed through the “event horizon” and as a result appeared to have experienced what Heidegger described as an authentic homecoming; they were at peace.

## Discussion

This paper explored subjective lived experiences of six individuals living in ExtraCare. It has illuminated pluralities within the experience of ageing through recognition of the co-existence of well-being and ill-health or incapacity, supporting Baars and Phillipson's (2013: 26) call for ageing to be understood in terms of “the potentials *and* limitations, the pleasures *and* sufferings, the continuing vitality, competence *and* vulnerability.” The experiential accounts analysed illustrate the significance of accepting the feeling of becoming aged, represented by overcoming the desire to focus on “being busy” and contentedly replacing it with “being” or “letting be” (Baars, 2012; Galvin & Todres, 2013). This is represented as peacefulness of mood (see Table I), an acceptance and coming to terms with things. To achieve dwelling-mobility, a sense of mirror-like multidimensional fullness, this would need to be accompanied by an energy to move forwards. This may include looking forward to a future event such as a community talent show or indoor bowls competition. Thus, the organization of such events in extra care communities can help create heightened well-being. This might be accompanied by a sense of ontological security (or layered continuity), a sense of self (identity) that exhibits a continuous sense of “I can” alongside a strong sense of being satisfied with “just being.” However, this state of ontological security was not in sight for some. Seeking care was perceived as an expression of dependency signifying lost autonomy (Fine & Glendinning, 2005), which could not be uncoupled from the “felt stigma” (Scambler, 1998) of unfulfilled marital duty or the embarrassment of personal care. Alongside these stories, though, was a case of care being used as an “optimizing strategy” (Baltes, 1996) facilitating engagement in social and imaginative pursuits, thus helping to retain a sense of independence while living with decreased capacity. In other words, a feeling of grounded vibrancy in the domain of embodiment: an energized flow toward being able to engage in activities and a sense of at-homeness with one's body.

Locating this analysis in the lifeworlds of individuals has helped turn the focus to the person-in-context. It was clear from Clive's butchers imagery, and Derek's weakness and increasing fatigue, that bodily changes were felt intensely as an existential challenge (Gilleard & Higgs, 2010). These participants’ experiences resonated with the existential angst of Heidegger's (1927) [1962)) “homelessness.” Sharing these feelings may help residents come to terms with them. Merleau-Ponty (1945) [1962]) believed that we are able to empathize with others’ bodily vulnerabilities more easily than we may think. Acknowledging this shared bodily vulnerable heritage would help residents - and staff - empathize with each other more readily. Lifeworld-led care (Galvin & Todres, 2013) takes up this concept of embodied knowledge arguing that the capacity to care requires the integration of epistemology (knowing), ontology (being) and ethics (what is good) echoing Walseth and Schei's (2011) call to embrace both the system world and the subjective lifeworld when making decisions about what is feasible, right, and good for those in receipt of care. In practice this approach calls for open dialogue and “empathic imagination” which actively engages the individual and care provider in imaginatively thinking through potential outcomes and the particularities of the individual's lifeworld in order to achieve dwelling-mobility across the domains of the lifeworld (Galvin & Todres, 2013). For example incorporating emotional support into consultations about care needs may help residents come to accept seeking care as an “optimizing strategy” (Baltes, 1996). An alternative may be to develop a peer mentoring model where residents support each other, thus fostering a sense of kinship and possibility within the intersubjective dimension. This would require training for residents but has been shown to be effective (e.g., Dorgo, King, Bader & Limon, 2013).

Supporting Machielse and Hortulanus (2013), our findings have highlighted the importance of achieving a balance between engagement in meaningful activities and social networks while maintaining autonomy, independence and personal choice. Social connectedness provides companionship but that must be on terms acceptable to the individual. Annie's and Martha's accounts indicated the potential difficulties in negotiating new relationships and getting involved in activities hosted by ExtraCare. Furthermore, the enjoyment of quiet activities like reading and puzzles suggested the need to extend the range of activities available to accommodate more residents; not everyone is a performer like Eleanor. Edgar's love of spending time with his neighbours’ pets also demonstrated how low key, “neighbourhood” events within small sections of the community might help encourage some residents to take up opportunities for social interaction. ExtraCare is dependent on volunteers to organize and host such enrichment activities. Smaller, more informal events may alleviate some of this pressure.

There is a need to acknowledge more openly residents’ being-towards-death (Heidegger, 1927 [1962]), i.e., their experience of becoming aged and their readiness for death. Temporality is a powerful element of the lifeworld and achieving a sense of renewal (see Table I) in the context of threats to well-being takes effort. Dwelling-mobility encompasses being absorbed in the now and a welcome readiness for the future. In the context of ageing this requires genuine anticipation of death (Moran, 2000). Derek was beginning to come to terms with his agedness through his growing fatigue and “winding down.” Edgar, too, expressed a sense of life lived, an acceptance of his fate. Further research about agedness and end of life in old age, outside of the context of diagnosed terminal illness, is needed to fully appreciate the meanings it holds. What this study shows is that individuals can reach a place where they accept that the future is short, where they can “let be,” feel secure in themselves, and experience a sense of well-being. This peaceful well-being might represent the authentic homecoming described by Heidegger (1927) [1962)) and works to prepare an individual for the last act of life, death. By focusing on the lived experience of ageing, we are able to have a dialogue with theoretical formulations of agedness to explore what it means for a person's well-being. Taking an existential–phenomenological approach in research to understand in-depth individuals’ lived experience of ageing in ExtraCare has provided an alternative interpretation which prioritizes the person, echoing the person-centred model dominant in this sector of care. It is also a methodology which is commensurate with healthcare practice through the use of practical wisdom and empathic imagination (Galvin & Todres, 2013).

Of course this study is situated within one UK extra care organization. Further interpretative research investigating the phenomenon of ageing from the perspective of older adults is required to more fully understand the complexities within it. More work is required to understand experiences of ageing in different kinds of assisted living environments. In addition, the impact of different kinds of service provision on individuals’ lifeworlds is necessary before policy recommendations can be made. In our larger study, the extra care housing model has been shown to be protective at the individual level in terms of physical and mental health outcomes, producing savings in relation to access to primary and secondary national healthcare services and social care costs (Holland et al., 2015).

This paper reported a phenomenological study about the experience of living, and ageing, in ExtraCare. By adopting an idiographic, interpretative approach we have been able to describe in-depth what it is like to live in ExtraCare. We have identified that an authentic later life is possible but individuals need support to navigate their path through the existential challenge of becoming aged. Hence, as well as providing enrichment activities and healthcare, extra care housing needs to provide emotional support to enable individuals to become ontologically secure and to live well to the end.

## Footnotes

1 All names are pseudonyms and identifying information has been removed from verbatim data extracts.
